# Duct ligation/de-ligation model: exploring mechanisms for salivary gland injury and regeneration

**DOI:** 10.3389/fcell.2024.1399934

**Published:** 2024-06-25

**Authors:** Bin Wang, Zhilin Li, Wei An, Gaiping Fan, Dezhi Li, Lizheng Qin

**Affiliations:** ^1^ Department of Head and Neck Oncology, Shanxi Province Cancer Hospital, Shanxi Hospital Affiliated to Cancer Hospital, Chinese Academy of Medical Sciences, Cancer Hospital Affiliated to Shanxi Medical University, Taiyuan, China; ^2^ Department of Oral and Maxillofacial Surgery, Shanxi Provincial People’s Hospital, Shanxi Medical University, Taiyuan, China; ^3^ Department of Head and Neck Oncology, National Cancer Center, National Clinical Research Center for Cancer, Cancer Hospital, Chinese Academy of Medical Sciences and Peking Union Medical College, Beijing, China; ^4^ Department of Oral and Maxillofacial and Head and Neck Oncology, Beijing Stomatological Hospital, Capital Medical University, Beijing, China

**Keywords:** salivary gland, duct ligation/de-ligation, injury, regeneration, animal model

## Abstract

Sialadenitis and sialadenitis-induced sialopathy are typically caused by obstruction of the salivary gland ducts. Atrophy of the salivary glands in experimental animals caused by duct ligation exhibits a histopathology similar to that of salivary gland sialadenitis. Therefore, a variety of duct ligation/de-ligation models have been commonly employed to study salivary gland injury and regeneration. Duct ligation is mainly characterised by apoptosis and activation of different signaling pathways in parenchymal cells, which eventually leads to gland atrophy and progressive dysfunction. By contrast, duct de-ligation can initiate the recovery of gland structure and function by regenerating the secretory tissue. This review summarizes the animal duct ligation/de-ligation models that have been used for the examination of pathological fundamentals in salivary disorders, in order to unravel the pathological changes and underlying mechanisms involved in salivary gland injury and regeneration. These experimental models have contributed to developing effective and curative strategies for gland dysfunction and providing plausible solutions for overcoming salivary disorders.

## 1 Introduction

Sialadenitis and sialadenitis-induced sialopathy are typically caused by obstruction of the salivary gland ducts (Mandel, 2014; Abdel Razek and Mukherji, 2017). Inflammation, trauma, and salivary stones are the most common causes of salivary gland obstructions, which can result in tissue damage and diminished secretory function of the gland, impacting a patient’s quality of life (Singh, et al., 2012; Pedersen, et al., 2018; Moorthy, et al., 2021). Generally, the salivary gland is preserved in patients with short-term obstruction, no recurrent gland infection, and normal operation of the gland. However, the salivary glands are typically removed when long-term duct obstruction leads to irreversible atrophy of the glands, as there is little to no chance of functional recovery (McGurk and Brown, 2009; Beneng, 2022). At present, a regenerative approach is expected to be an effective way to treat salivary gland diseases and repair damage, but it is yet to be clinically approved and widely used in human patients (Chibly, et al., 2022). Salivary gland duct ligation/de-ligation are used to model and study the processes of glandular dysfunction, injury, and subsequent regeneration, providing valuable insights for developing therapeutic interventions for salivary gland disorders (Kim, et al., 2023; Yang, et al., 2023). The compiled animal duct ligation/de-ligation studies indicate that the most common animals used to create ductal obstruction models are mice, rats, cats, and rabbits. In this review, we aim to provide suggestions for salivary gland regeneration and treatment of salivary gland disorders by summarising the characteristics and mechanisms of the animal duct ligation/de-ligation model. The results obtained from the animal models may prove beneficial to both researchers and clinicians, supporting preclinical research for the future development of novel therapeutic techniques for salivary gland hypofunction.

## 2 Salivary gland duct ligation

Duct ligation-induced atrophy of the salivary glands in experimental animals displays a histopathology that resembles that of human diseases (Kim, et al., 2023). Salivary gland duct ligation modelling is mainly performed in the submandibular gland (SMG) and less in the parotid gland (PG) (Table 1). This may be because the SMG is the most well-studied gland in mice, and duct ligation is far easier to study in the SMG than PG due to the single major duct that empties the contents of the SMG into the oral cavity, while the PG has multiple ducts. The duration of the obstruction varied considerably from 1 h to 1 year, which could be appropriate for reproducing acute and chronic sialadenitis and fibrosis (Harrison, et al., 2000; Correia, et al., 2008). Both human salivary gland duct obstruction and animal salivary gland duct ligation caused noticeable morphological changes in the acinar and ductal cells, leading to gland atrophy and progressive dysfunction (Stojanov, et al., 2017).

**TABLE 1:** The animal salivary gland duct ligation model in previous studies.

Authors (Year)	PMID	Animal	Ligated gland	Control gland	Ligated time	Materials and methods	Purpose
Takai et al. (1985)	3,923,174	ddY mice/male and female	Right SMG (unilateral)	Contralateral	1, 2, 3, 6, 12 h/1, 2, 3, 4, 5, 7, 10, 14 days	Extra-oral/4–0 silk suture	To detect the expression of EGF and NGF on immunohistochemistry in duct-ligated mouse SMG.
Zaia et al. (1996)	9,021,337	C57BL/6 mice/male	Right SMG (unilateral)	Contralateral	2, 8, 14, 28 days	Extra-oral/cotton thread	To study the changes that occur in the expression of collagen and elastic fibres during experimental atrophy of the SMG in mice
Ohno et al. (2007)	17,305,616	BALB/c mice/female	Right SMG (unilateral)	Normal	8 weeks	Extra-oral/silk thread	To investigate the effect on the onset of autoimmunity against the salivary gland parenchyma with reference to genetic background
Purwanti et al. (2011)	21,868,636	C57BL/6 mice/male	Left SMG (unilateral)	Contralateral	0, 1, 3, 6, 12, 24 h/1, 3, 6 days	Extra-oral/surgical suture	To examine the initial step that takes place immediately after tissue injury and is linked to tissue regeneration
Bozorgi et al. (2014)	24,675,464	ICR mice/female	Left SMG (unilateral)	Normal	3, 5, 7 days	Extra-oral/6–0 Ethicon suture	To investigate the effects of mTOR inhibition on duct ligation-induced salivary gland atrophy
Takahashi et al. (2017)	29,070,775	C57BL/6 J mice/male	Right SMG (unilateral)	Sham	1, 3, 7 days	Extra-oral/7–0 braided silk suture	To analyse the expression of Ror2 associated with fibrosis of the SMG.
Zhang et al. (2022)	36,216,809	C57BL/6 mice/female	SMG (unilateral)	Sham	2 weeks	Extra-oral/micro-titanium clip	To elucidated the essential role of IFT140 and cilia in regulating salivary stem/progenitor cell differentiation and gland regeneration
Li et al. (2023)	37,021,213	C57BL/6 J mice/male	SMG	Sham	7 days	Extra-oral	To analyse the effects of SIS3 on the SMG dysfunction, fibrosis, and inflammation
Mao et al. (2023)	36,112,881	C57BL/6 mice/male	Right SMG (unilateral)	Sham	1, 3, 7 days	Extra-oral/surgical suture	To explore the alteration and contribution of endothelial TJs in the fibrosis of the SMG from a mouse duct ligation model
Tamarin (1979)	119,842	Sprague-Dawley rats/male	Left SMG (unilateral)	Contralateral	1h to 31 days	Extra-oral/a single strand of stainless-steel wire/2 mm from the hilum of the gland	The purpose of the report is to provide a description of the leukocytic response as seen in experimental obstruction of the rat SMG.
Walkeret al. (1987)	3,430,235	Sprague-Dawley rats/male	Right PG (unilateral)	Normal	12, 18 h/1, 2, 3, 4, 5, 7 days/2, 3, 4, 8, 12, 24 weeks	Extra-oral/4–0 silk (doubly ligated)	To analyse the cell death and cell proliferation during atrophy of the rat PG induced by duct obstruction
Martinez et al. (1982)	6,956,256	Sprague-Dawley rats/male	SMG (unilateral)	Contralateral	1, 3, 7, 14 days	Extra-oral/triple-O surgical suture/distal to the gland hilum	To explore secretory function in the rat SMG after excretory duct ligation
Hashimoto et al. (1992)	1,383,500	Wistar rats/male	Right SMG (unilateral)	Contralateral	3, 6, 10, 14, 21 days	N.A.	To assess the biologic significance of S-100 protein and rEGF in granular ductal cells in duct-ligated SMGs
Miguel et al. (2002)	12,945,732	Wistar rats/male	PG	Normal	1, 7, 15, 21, 30, 60 days	Extra-oral/cotton suture	To study the temporal expression of calponin in myoepithelial cells of the ligated rat PG.
Takahashi et al. (2000)	11,197,228	Wistar rats/male	Right SMG (unilateral)	Normal	1, 2, 3, 4, 5, 7, (10), 14, (21), 28 days	Extra-oral/metal clip (doubly ligated)	To clarify the role of parenchymal cell apoptosis and mitosis during atrophy of rat SMG induced by double ligation
Takahashi et al. (2001)	11,724,903	Wistar rats/male	Right SMG (unilateral)	Normal	1, 2, 3, 4, 5, 7, (10), 14, (21), 28 days	Extra-oral/metal clip (doubly ligated)/near the hilum of the gland	To determine whether apoptosis and proliferation of myoepithelial cells occur in atrophic rat SMG.
Takahashi et al. (2007)	17,244,334	Wistar rats/male	Right SMG (unilateral)	Normal	1, 3, 5, 10, 14 days	Extra-oral/ligaclips (doubly ligated)/near the hilum of the gland	To elucidate whether Fas and its receptor ligand (FasL) are involved in apoptotic atrophy of the salivary glands
Takahashi et al. (2008)	18,808,524	Wistar rats/male	Right SMG (unilateral)	Normal	1, 3, 5, 10, 14 days	Extra-oral/ligaclips (doubly ligated)/near the hilum of the gland	To evaluate whether Bcl-2 and Bax regulating the signaling pathway of apoptosis were involved in ductal cell survival and acinar cell death in atrophic SMG.
Correia et al. (2008)	18,221,457	Wistar rats/male	Right SMG (unilateral)	Contralateral	1 day	Intra-oral/metal micro-clip/posterior to the ductal orifice	To explore relationship between the salivary hypofunction and inflammation caused by duct ligation
Silver et al. (2010)	20,890,458	Wistar rats/male	Right SMG (unilateral)	Normal	1, 3, 5, 7, 9, 14 days	Intra-oral/metal micro-clip/5 mm posterior to the ductal orifice	Activation of mTOR coincides with autophagy during ligation-induced atrophy in the rat submandibular gland
Jung et al. (2011)	20,926,064	Sprague-Dawley rats/male	Right SMG (unilateral)	Normal	1, 3, 7, 14, 21 days	Extra-oral	To elucidate the ENaC expression in excretory duct obstruction of the SMG.
Hishida et al. (2016)	27,303,108	Wistar rats/male	SMG	Normal	1, 3, 5, 7 days	Extra-oral/3–0 (8–0) silk sutures/mid portion/the orifice of the duct	To analyse the expression of SPFs and SPRs in the atrophy of the SMG.
Yang et al. (2023)	36,950,625	Wistar rats/male	Left SMG (unilateral)	Normal	1 day/1, 2, 3, 4 weeks	Extra-oral/non-absorbable suture	To explore the histopathological and genetic changes in the SMG after duct ligation and provide important clues to functional regeneration
Maria et al. (2014)	24,053,197	New Zealand rabbits/male	PG (bilateral)	Sham	1, 7, 14, 30, 60 days	Extra-oral/3–0 silk suture (doubly ligated)	To elucidate the effects of long term, unrelieved main duct ligation on the rabbit PG.
Li et al. (2023)	30,125,980	Japanese white rabbits	SMG (unilateral)	Sham	2, 4, 8 weeks	Extra-oral/6–0 nylon thread/at 5 mm behind the orifice of the duct	To clarify the morphological change of the ligated rabbits SMG.
Harrison et al. (2000)	1,271,136	Cats/male and female	PG, SMG, SLG (unilateral)	Contralateral	From 1 to 365 days	Extra-oral/3–0 braided silk	To study the histological effects of duct ligation of salivary glands of the cat
Harrison et al. (2000)	11,000,380	Cats	PG (unilateral)	Contralateral	From 1 to 365 days	Extra-oral/3–0 silk suture	To examine the changes of acinar cells in the cat ligated PG.

PG, parotid gland; SMG, submandibular gland; SLG, sublingual gland.

### 2.1 Structure and function in salivary gland duct ligation

Salivary glands consist of glandular secretory tissue (the parenchyma) and supporting connective tissue (the stroma). The parenchyma is composed of ducts and secretory acini. The morphology of salivary gland tissue frequently appears lobulated and well-defined under normal conditions (Amano, et al., 2012). The main pathological characteristics of salivary gland atrophy are degeneration, disappearance of the acinus, expansion and proliferation of duct-like structures, infiltration of inflammatory cells, and interstitial fibrosis (Porcheri and Mitsiadis, 2019). However, variations in the timing of duct ligation can affect the extent of gland injury (Maria, et al., 2014). In rats, the SMG exhibits significant neutrophil infiltration within 18 h post-duct ligation and obvious monocyte infiltration within 24 h (Tamarin, 1979; Norberg, et al., 1988). Generally, the stimulated salivary flow rate of the ligated glands was significantly reduced to only 30.6% of that of the normal salivary glands. However, there are some limitations in measuring the salivary flow rates after duct ligation. Firstly, ligation of a single SMG may have little impact on the whole salivary flow because the salivary flow depends on saliva secreted by the three major salivary glands. Secondly, the SMG is the major contributor to resting saliva, and the PG is the major contributor to stimulated saliva; the variation in stimulated saliva after a single SMG ligation is not readily apparent (Miranda-Rius, et al., 2015). Additionally, protein secretion and ion reabsorption of the ligated glands, which contain higher concentrations of sodium and chloride ions in the saliva, are also seriously impaired. Electron microscopy revealed sparse pools of endoplasmic reticulum in most acinar cells, and the cytoplasm was filled with secretory granules that appeared to be fused. Biochemical analysis showed that ductal kinase also decreased by 50%, indicating that the reabsorption function of salivary gland ducts may be significantly inhibited by 24 h post-duct ligation (Carpenter, et al., 2007; Correia, et al., 2008).

Consequently, short-term ligation can lead to significant disturbances in the structure and function of the salivary glands. At 48 h post-ligation, the secretory function and weight of the gland steadily decreased as gland atrophy became more apparent, accompanied by grievous degranulation of secretory cells (Osailan, et al., 2006). Seven days post-duct ligation, atrophy of the acinar and duct of the ligated glands was evident, and macrophages and inflammatory cells in the lobules were still present in large numbers (Martinez, et al., 1982). Currently, 7-day duct ligation is the most commonly used model for studying salivary gland regeneration. However, 14 days post-duct ligation, significant fibrosis was observed around the acinus and ducts, with the appearance of adhesive material within the ducts, resulting in a 96.8% reduction in salivary secretion. The concentration of sodium in the saliva gradually increased to the same level as that in the plasma. Furthermore, there was a significant decrease in the quantity of cholinergic and adrenergic receptors and a notable reduction in the secretory response to phenylephrine and isoproterenol. Subsequently, 14-day duct ligation has become the most frequently employed model for the research of salivary gland fibrosis (Miyazaki, et al., 2008).

Two months after SMG duct ligation, histological analysis shows that the majority of acinar tissue had disappeared, interlobular connective tissue hyperplasia and fibrosis were evident, and only the remaining part of the duct-like structure was present. These findings are in line with those of short-term ligation studies. To date, the longest period of experimental obstruction of the main SMG duct is 2 months (Watanabe, et al., 2017). However, Harrison et al. performed a 365-day ligation of the cat PG duct and found that the glandular tissue was almost completely replaced by fibrous tissue, while some acinar cells were still present and preserve typical acinar cell ultrastructural characteristics, with the exception of reduced secretory granules and small cell size (Harrison, et al., 2000). Therefore, long-term ligation eventually leads to shrinkage and fibrosis of the glands but does not cause the glands to completely atrophy and disappear, which may be due to the differential responses between SMG and PG.

### 2.2 Mechanism of salivary gland duct ligation

**FIGURE 1:**
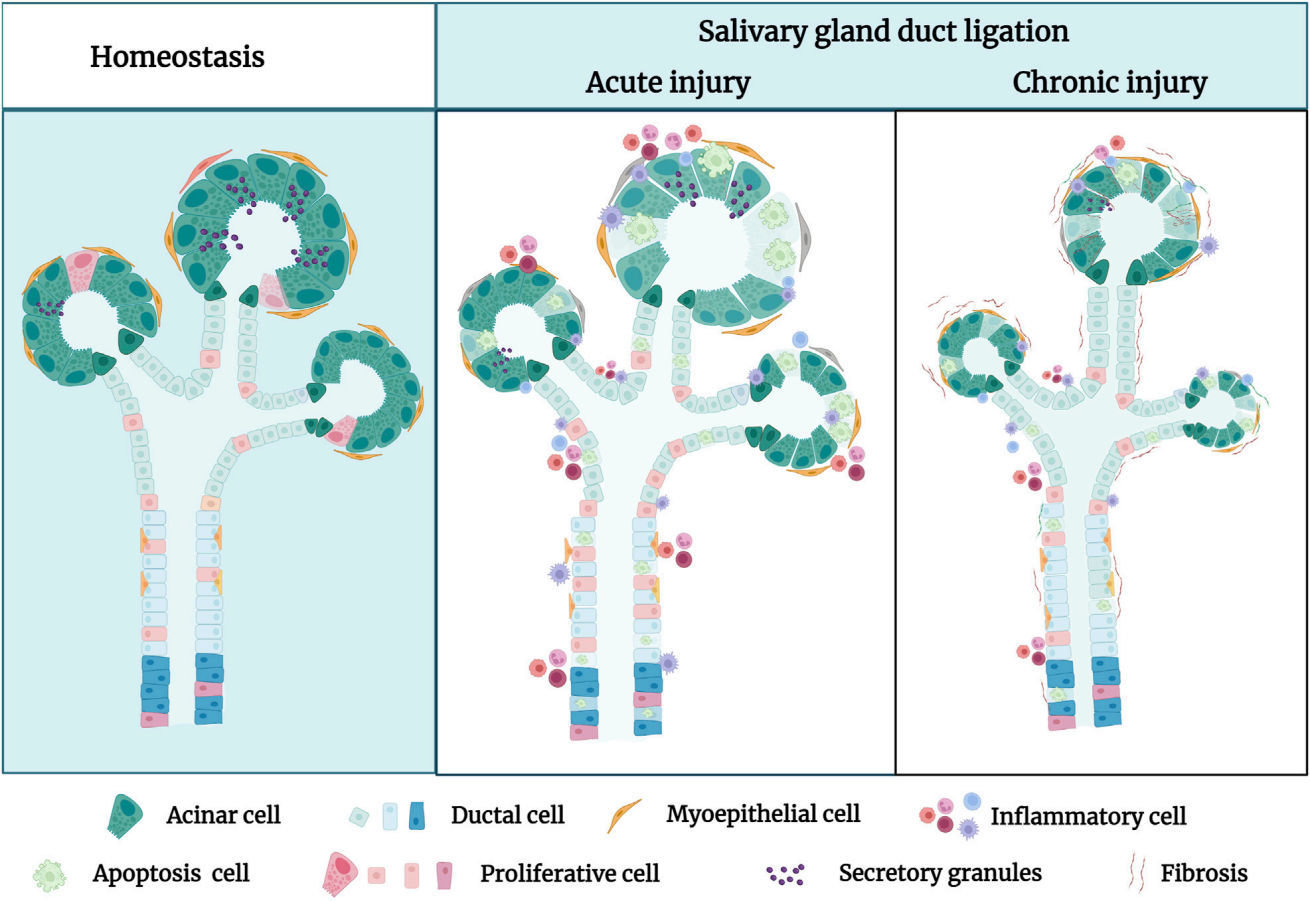
The pathological characteristics of the salivary gland in homeostatic condition and after duct ligation. In homeostasis, the morphology of secretory acini and ducts frequently appears lobulated and well-defined under normal conditions. In acute injury, the primary manifestations of the salivary gland after duct ligation are acinar cell apoptosis, duct dilation, inflammatory cell infiltration, and decreasing secretory granules. In chronic injury, the secretory acini and ducts of salivary gland are significantly atrophied, and the glandular tissue is almost gradually replaced by fibrous tissue after long-term duct ligation. Figure was created with BioRender.com.

In a duct-ligated salivary gland model, experimental studies demonstrated that the apoptosis and proliferation of parenchymal cells are affected (Burgess and Dardick, 1998). Duct ligation induces a substantial apoptotic loss of acinar cells and concurrent brief cycle of ductal cell proliferation (Figure 1). Walker and Gobe demonstrated the rapid disappearance of acinar cells via apoptosis 24 h after rat PG duct ligation (Walker and Gobe, 1987). Takahashi, et al., 2000 demonstrated the presence of a large number of acinar cells and occurrence of partial ductal cell apoptosis during the atrophy of the duct-ligated rat SMG, in which the apoptotic rate of terminal deoxynucleotidyl transferase dUTP nick end labelling (TUNEL)-positive acinar cells reached 18.4%, whereas that of TUNEL-positive ductal cells was only 3%. Furthermore, electron microscopy revealed that a large number of acinar cells and some ductal cells showed ultrastructural changes during apoptosis, especially the chromatin-dense group adjacent to the nuclear envelope, with obvious boundaries. Apoptotic bodies are observed in the cytoplasm of glandular epithelial cells and can be engulfed and degraded by intraepithelial macrophages or neighbouring acinar cells. A significant ductal cell mitotic rate and proliferating cell nuclear antigen (PCNA) labelling index were noted 2 and 3 days after PG and SMG duct ligation. After a 7-day duct ligation, the gland displayed a discernible decrease in the quantity of acinar cells and corresponding increase in duct volume. The higher duct quantity seen in atrophic glands could be attributed to duct expansion (Takahashi, et al., 2002). Both epithelial and myoepithelial cells undergo apoptosis and proliferate during a gland ligation injury. Burgess et al. examined rat PG that had undergone duct ligation-induced atrophy and discovered that both atrophied and normal PG had proliferating myoepithelial cells (Burgess, et al., 1996). Takahashi et al. also demonstrated the presence of PCNA-positive myoepithelial cells surrounding the ducts in atrophic SMG, suggesting that myoepithelial cells possess active proliferative abilities. The maximum PCNA index of the myoepithelial cells in the atrophic PG (23.1%) was much higher than that in the atrophic SMG (7.55%) (Takahashi, et al., 2001). However, there was a distinction in myoepithelial cell apoptosis between the atrophic PG and SMG; no sign of myoepithelial cell apoptosis was observed in the latter through transmission electron microscopy (TEM). Thus, rapid apoptotic deletion of acinar cells and slow proliferation are the predominant mechanisms in duct-ligated exocrine glands (Takahashi, et al., 2004a). However, as the ligation time increases, proliferation gradually decreases or even disappears, manifesting as apoptosis. Further research is necessary to uncover the mechanisms of apoptosis and proliferation.

### 2.3 Signaling pathways of salivary gland duct ligation

Studies indicate that duct ligation induces apoptosis and proliferation of gland cells by activating various intrinsic and extrinsic signaling pathways. These signals activate signaling cascades, resulting in cell shrinkage and cytoskeletal disruption (de Mello Gomes, et al., 2019). Takahashi et al. suggested that Bcl-2 and Bax are involved in the apoptotic signaling of atrophied SMG after excretory ducts were ligated for 14 days. Immunohistochemical analysis showed strong expression of Bax and Bcl-2 was respectively detected in acinar and ductal cells, respectively, after ligation. Meanwhile, RT-PCR analysis showed Bcl-2 mRNA expression increased stronger as the glandular atrophy progressed, which may indicate that Bcl-2 contributes to the survival of ductal cells. In comparison, the expression of Bax mRNA increased after 1 and 3 days, which may indicate that Bax accelerates acinar cell apoptosis in the atrophy of SMG (Takahashi, et al., 2008).

Fas and FasL signals are tightly associated with the induction of apoptosis in atrophied SMG. Fas triggering strongly activates caspase 3/8 and induces morphological changes associated with cell apoptosis and chromosomal DNA degradation (Takahashi, et al., 2007). Multiple researchers have suggested that the Fas/FasL system is involved in the acinar and ductal cell apoptosis of Sjögren’s syndrome (SS) (Shibata, et al., 2002; Hayashi, et al., 2003). Woods et al. showed that the activation of P2X7R in the SMG induces an aggravated inflammatory response and promotes apoptosis after duct ligation. ATP or high-affinity P2X7R agonist 3′-O-(4-benzoyl) benzoyl-ATP (BzATP) treatment not only increased the infiltration of immune cells in the glands after ligation but also led to the formation of protrusions in the cell membrane and activated caspase, ultimately leading to apoptosis of glandular epithelial cells (Nakamoto, et al., 2009; Woods, et al., 2012). Furthermore, Khalafalla et al. demonstrated that P2X7R antagonism limits gland inflammation and improves secretory function, representing an innovative therapeutic approach for preventing human salivary gland inflammatory disorders (Khalafalla, et al., 2017).

**FIGURE 2:**
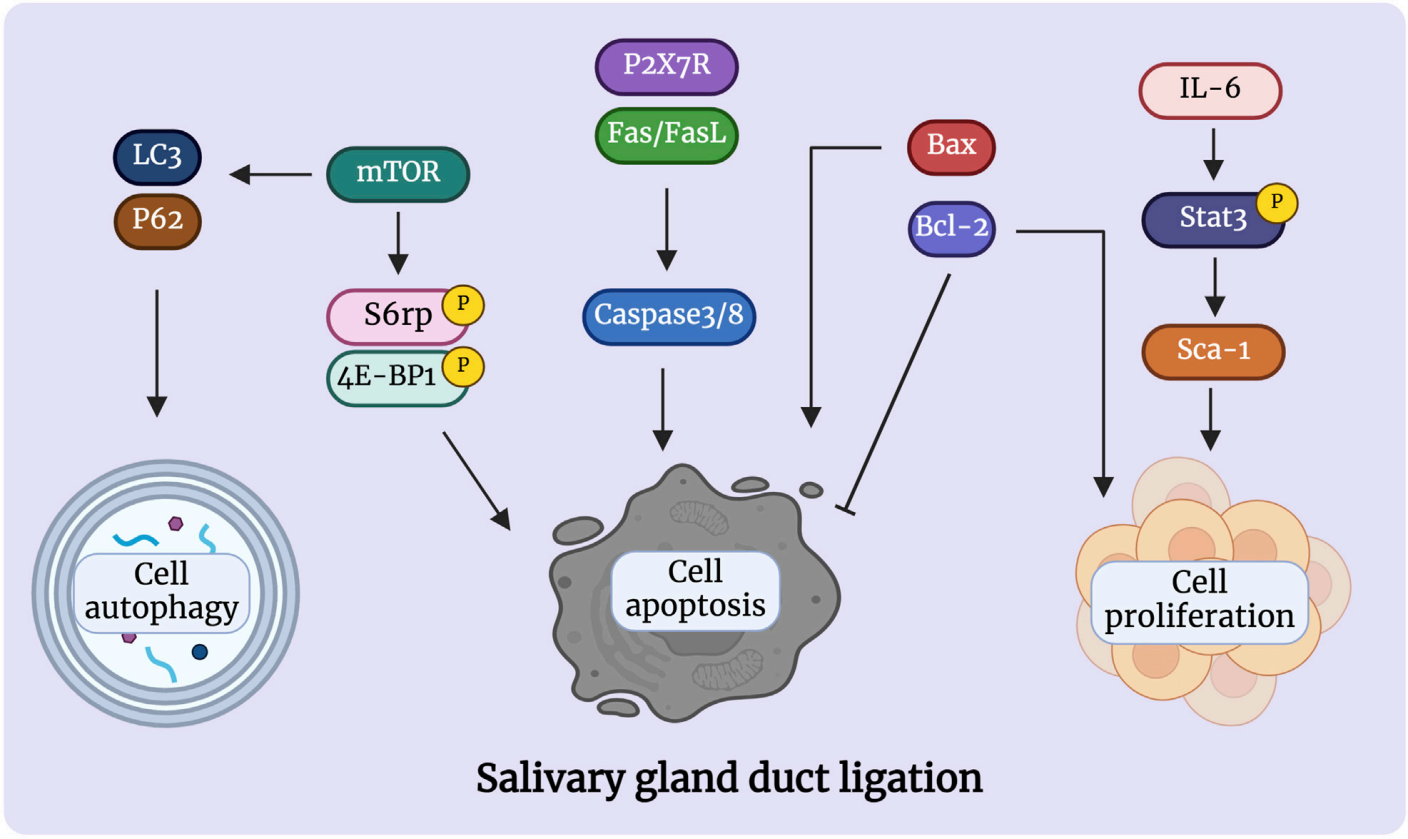
Signaling pathways are involved in the atrophy process after salivary gland duct ligation. Duct ligation can induce apoptosis and proliferation of gland cells by activating various intrinsic and extrinsic signaling pathways. The proteins Bcl-2, Bax, Fas/FasL, and P2X7R are detected in the acinar and ducts govern cell apoptosis during duct ligation. Activation of mTOR can regulate apoptosis and autophagy, which may play an important role in the preservation of the remaining acinar cells. Activation of Il-6/Stat3/Sca-1 signaling may be responsible for short-lived ductal cell proliferation after duct ligation. Figure was created with BioRender.com.

Bozorgi et al. discovered that mouse SMG duct ligation promotes mTOR activation, which affects protein synthesis and controls cell apoptosis. Rapamycin prevented the atrophy of salivary glands caused by ligation by inhibiting mTOR. The ligated glands treated by rapamycin produced larger acinar cells than normal glands but had no discernible effect on inflammatory cell infiltrates (Correia, et al., 2008; Cotroneo, et al., 2008; Bozorgi, et al., 2014). Silver et al. found that duct ligation regulates the activation of mTOR, which controls cell autophagy (Silver, et al., 2010). Electron microscopy has been used to detect autophagosomal vesicles during ligation-induced gland atrophy (Tamarin, 1971; Hand and Ball, 1988). Although the evidence for autophagy in salivary gland ligation has been previously demonstrated, the function of autophagy has not been studied. The simultaneous activation of apoptosis and autophagy via mTOR may be an important mechanism for the preservation of the remaining acinar cells during salivary gland atrophy (Takahashi, et al., 2000). Thus, it is hypothesised that these two pathways have a strong correlation and a substantial degree of coordination with respect to the development of salivary gland atrophy and survival. However, Purwanti et al. showed that IL-6/STAT3 signaling peaks at 6 h after duct ligation and that the increase in the level of this inflammatory signaling triggers Sca-1 activation (Purwanti, et al., 2009). Through duct ligation, Sca-1 was strongly expressed in most cells of the striated duct and granular convoluted tubules, implying that Sca-1-positive cells may play a role in ductal cell proliferation during the regeneration step elicited by duct ligation-induced injury (Purwanti, et al., 2011) (Figure 2). Additionally, Hashimoto et al. showed that the negative expression of epidermal growth factor (EGF), nerve growth factor (NGF), and S-100 protein in duct obstructions leads to a reduction or elimination of the function of granular ductal cells. The loss of S-100 and EGF expression after ligation further indicated that the granular duct had lost its ability to repair, resulting in decreased tissue regeneration (Takai, et al., 1985; Takai, et al., 1986; Hashimoto, et al., 1992). Few studies have been conducted on salivary gland duct ligation injury to date, and it is unclear which signaling pathway is activated. Therefore, further research aiming to identify the underlying signals that regulate salivary gland injury is required.

## 3 Salivary gland duct de-ligation

Salivary glands possess the remarkable ability to recover by regenerating secretory tissue after duct de-ligation, partially restoring the structure and function of the gland. This involves the interactions of multiple cell types, including acinar, ductal, myoepithelial, and salivary gland epithelial stem/progenitor cells. Diverse pathways work together to develop acinar and duct development and cell survival/proliferation. Multiple studies have revealed that molecular pathways function inside complicated signaling networks but do not describe in detail how these pathways impact regenerative processes (Table 2).

**TABLE 2:** The animal salivary gland duct ligation/de-ligation model in previous studies.

Authors (Year)	PMID	Ligated gland	Control gland	Ligated time	De-ligated time	Materials and methods	Purpose
Ikai et al. (2020)	32,748,407	PG	Sham	1, 3 days	0, 7, 14 days	Extra-oral/silk thread	To elucidate the p63 expression in the regeneration process of the SG by duct ligation/de-ligation
Arany et al. (2011)	21,377,457	Left SMG (unilateral)	N.A.	10 days	7, 14, 21 days	Extra-oral/micro-clamp	To investigate the contribution of the Ascl3^+^ progenitor cell population to salivary gland maintenance and regeneration
Nagai et al. (2014)	2,435,788	Left SMG (unilateral)	Normal	7 days	0, 1, 3, 7, 14 days	Extra-oral/metal sugita titanium aneurysm clip	To investigate the roles of the EGF family during the course of salivary gland regeneration
Woods et al. (2015)	25,955,532	SMG (unilateral)	Contralateral	7 days	28 days	Extra-oral/surgical suture	To explore the expression of TGF-β signaling components relation to mouse SMG duct ligation-induced fibrosis and regeneration following duct de-ligation
Weng et al. (2018)	30,089,258	Left SMG (unilateral)	Contralateral	14 days	14 days	Extra-oral/titanium hemostatic clip	To investigate acinar cell replacement and regeneration during homeostasis and after duct ligation injury
Watanabe et al. (2017)	28,744,028	Right SMG (unilateral)	Contralateral	2 months	1 week/1, 2 months	Extra-oral/sugita titanium aneurysm clip/approximately 2 mm superior to the main body of gland	To analyze the regenerative capacity of the mouse SMG using an aneurysm clip duct ligation model
Ninche et al. (2020)	32,994,165	SMG (unilateral)	Normal	3 days	5, 10, 20 days	Extra-oral/6-0-black-silk suture	To assess the extent of injury and dynamics of recovery of acini in a model of severe and reversible injury
Rocchi et al. (2021)	34,874,744	SMG (unilateral)	Sham	14 days	30 days	Extra-oral/non-dissolvable wire	To identify a central role for YAP in the regenerative response of the salivary gland
Takahashi et al. (1998)	10,193,312	PG (unilateral)	Sham	7 days	0, 1, 2, 3, 4, 5, 7, 10, 14 days	Extra-oral/ligaclips (doubly ligated)	To examine the characteristic of acinar cells regeneration in the rat PG after atrophy induced by 1 week period of duct obstruction
Burgess et al. (1998)	9,638,704	PG (unilateral)	Normal	7 days	1, 5, 7, 10, 14 days	Extra-oral/3–0 silk	To explore the cell population changes during atrophy and regeneration of rat PG.
Burgess et al. (1996)	8,974,141	PG	Normal	7 days	1, 5, 7, 10, 14 days	Extra-oral/a V-shaped micro-clamp	To examine the proliferative capacity of myoepithelial cells after rat PG duct ligation/de-ligation
Takahashi et al. (1998)	10,607,019	Right PG (unilateral))	Normal	7 days	0, 1, 3, 5, 7, 10, 12, 14, 17, 21 days	Extra-oral/metal clip (doubly ligated)	To examine the myoepithelial cell changes during PG regeneration after complete duct obstruction induced by double ligation
Scott et al. (1999)	10,576,167	PG (unilateral)	Normal	1, 2, 4, 6 weeks	1, 2 weeks	Extra-oral/micro-clamp	To study the morphological and functional characteristics of acinar atrophy and recovery recovery in the duct ligated PG of the rats
Dang et al. (2008)	18,177,419	PG (bilateral)	Sham	7 days	7, 30 days	Extra-oral/3–0 silk ligature	To explore the role of MAPK signals in salivary acinar cell atrophy and regeneration
Ahn et al. (2000)	10,912,994	SMG (unilateral)	Contralateral	3 days	3, 7, 14, 21 days	Extra-oral/surgical suture/7 mm distal to the gland hilum	To investigate the reversible regulation of P2Y2 nucleotide receptor expression in the duct-ligated rat SMG.
Takahashi et al. (2004)	14,675,137	Right SMG (unilateral)	Normal	7 days	0, 1, 2, 3, 4, 5, 7, 10, 14 days	Extra-oral/metal clip (doubly ligated)/near the hilum of the gland	To clarify the proliferation and apoptosis of parenchymal cells during regeneration of rat SMG following atrophy
Takahashi et al. (2004)	15,250,836	Right SMG (unilateral)	Normal	7 days	1,2, 3, 4, 5, 7, (10), 14, (21), 28 days	Extra-oral/metal clip (doubly ligated)/near the hilum of the gland	To elucidate whether myoepithelial cells proliferate mitotically during regeneration of rat SMG after atrophy
Carpenter et al. (2007)	17,305,704	SMG (unilateral)	Contralateral	1 day	3 days	Intra-oral/metal clip/a small incision in the floor of the mouth	To investigate the effects of inflammation on rat SMG function following acute duct obstruction
Miyazaki et al. (2008)	18,507,618	Right PG (unilateral)	Sham	1, 2, 3, 6, 14 days	2, 8, 14, 21, 25, 28 days	Extra-oral/a clip	To examine the proliferative process of rat acinar cells after PG ligation and reopening
Cotroneo et al. (2008)	18,335,244	SMG (unilateral)	Normal	2 weeks	3 days	Intra-oral/metal clip	To identify the earliest morphological and molecular markers of regeneration occurring in rat SMG after intra-oral ligation
Ueda et al. (2009)	19,097,859	SMG (bilateral)	Normal	7 days	0, 1, 3, 5, 7, 11 days	Extra-oral/metal clip/near the hilum	To determine the localization of tenascin-C, fibronectin and collagen types III and IV during regeneration of the rat SMG.
Carpenter et al. (2009)	11,724,903	Right SMG (unilateral)	Contralateral	4 weeks	8 weeks	Intra-oral/metal clip/a small incision in the floor of the mouth	To explore the role of parasympathetic innervation in the recovering rat SMG following extensive atrophy
Mizobe et al. (2015)	25,859,059	Right SMG (unilateral)	Contralateral	1, 3, 7 days	1, 2 weeks	Extra-oral/surgical vascular ligation clip/5 mm distal to the glandular porta	To assess the relationship between Hsp27 and compensatory hypertrophy in salivary glands
Shimizu et al. (2015)	26,173,945	SMG (bilateral)	Normal	7 days	0, 1, 3, 7, 11, 14 days	Extra-oral/metal clip/near the hilum of the gland	To clarify the localizations and functions of FGFs and FGFRs during salivary gland regeneration
Yasumitsu et al. (2018)	30,587,691	SMG (bilateral)	Normal	7 days	0, 3, 7, 14 days	Extra-oral/metal clip	To investigate the distributions of AQP5, TGF-β1, and laminin and to clarify the environment around duct-like structure during regeneration
Wang et al. (2023)	35,593,110	Right SMG (unilateral)	Contralateral	7 days	0, 1, 3, 7, 14, 28 days	Extra-oral/SLC-CLIP titanium hemostatic clip	To elucidate the role of sialin in the salivary gland regeneration

PG, parotid gland; SMG, submandibular gland; N.A., not available.

### 3.1 Structure and function in salivary gland duct de-ligation

Studies indicated that the structure and function of the salivary glands can be fully restored after ligation/de-ligation. Ahn, et al., 2000 showed that the majority of acinar cells returned to normal after 3 days of duct ligation and 7 days of de-ligation; however, there were some abnormalities in the duct system, manifesting as fewer secretory granules in the granular duct under electron microscopy. Fourteen days after de-ligation, the morphology and function of the atrophic glands were completely restored. Takahashi et al. found that the endoplasmic reticulum cisterna was regularly arranged in layers around the nucleus with the maturation of acinar cells, and the cytoplasmic lumen contained a large number of zymogen granules after 7 days of duct ligation/de-ligation. Fourteen days after de-ligation, the BrdU-positive acinar cells and morphology of the SMG returned to those of the control group, suggesting that the morphology of the PG recovered after removal of the duct ligation (Takahashi, et al., 1998). Woods et al. revealed that structure and function could be fully restored 7 days after duct ligation and 28 days after de-ligation. The acinar cell marker AQP5 expression, the number of immune cells, and secretory granules in the ductal cells returned to a level comparable to that of the control glands on day 28 post-ligation (Woods, et al., 2015).

Most studies also demonstrated that the weight and the morphology are almost entirely recovered on day 28 post-de-ligation (Wang, et al., 2022). However, long-term duct obstruction may result in the permanent atrophy of salivary glands. Watanabe, et al., 2017 showed that when the SMG duct was ligated for 2 months, the gland was severely damaged, and the structure and function could not be fully restored after de-ligation; Long-term duct ligation may raise the risk of salivary gland infection. These results indicate that the gland retains a certain regenerative ability, albeit a limited one, after long-term duct occlusion. No definitive conclusions have been reached regarding the time it takes for complete glandular recovery post-duct de-ligation. Therefore, further research should investigate this under specific circumstances during duct ligation/de-ligation.

### 3.2 Mechanism of salivary gland duct de-ligation

**FIGURE 3:**
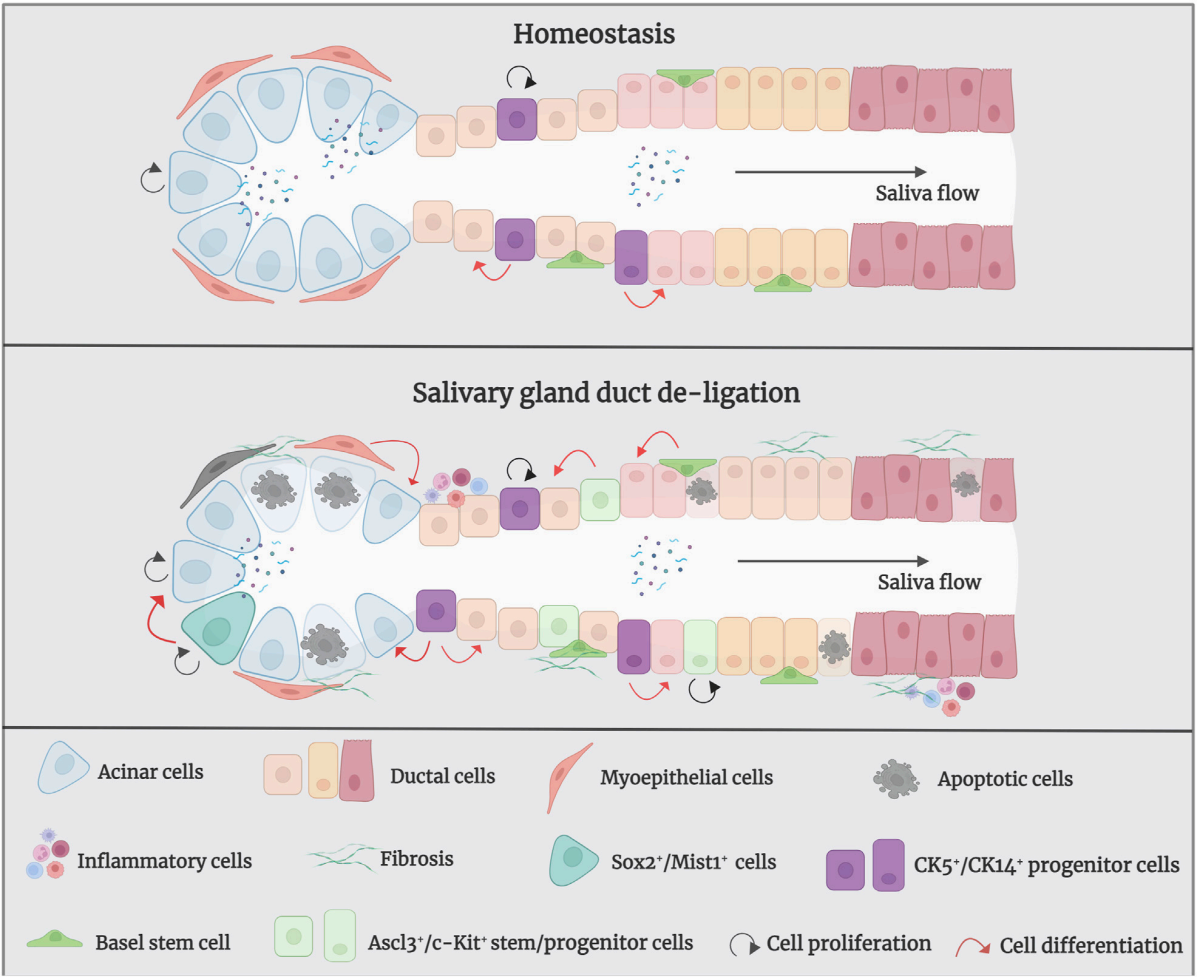
The mechanism of salivary gland regeneration after duct de-ligation. In homeostasis, ductal and acinar cells are lineage-restricted and maintained themselves separately. After duct ligation/de-ligation, both acinar cells and ductal cells can generate acini during the regeneration process. Myoepithelial cells restore the state of bipotent progenitor cells and subsequent re-differentiation into acinar cells. In addition, CK5^+^/CK14^+^/Ascl3^+^/c-Kit^+^ salivary gland stem/progenitor cells undergo differentiation into to acinar cells or ductal cells, which produce new acini. Sox2^+^/Mist1^+^ acinar cells also contribute to the production of acini after duct de-ligation. Figure was created with BioRender.com.

In salivary glands, replacement of ductal and acinar cells occurs through lineage-restricted progenitors under homeostatic conditions; acinar cells are replenished by self-duplication, and ductal cells are supplemented by cytokeratin 14-positive (CK14^+^) and cytokeratin 5-positive (CK5^+^) ductal progenitor cells in the adult mice SMG (Weng, et al., 2018). Within duct ligation/de-ligation models, it remains unclear whether acinar, ductal, or other parenchymal cells are responsible for repair and regeneration (Aure, et al., 2015a) (Figure 3). Weng et al. found that acinar and ductal cells replenish lost cells mainly through self-replication under normal homeostatic conditions; however, this entirely depends on the proliferation of the remaining surviving acinar cells and does not rely on ductal cells. The primary explanation for this is that the surviving acinar cells still have an extremely remarkable proliferative ability after de-ligation (Aure, et al., 2015b; Weng, et al., 2018). However, differentiating ductal cells into acinar cells after duct de-ligation has been suggested. Walker and Cobe demonstrated significant mitosis in the striated and intercalated ducts after rat PG duct de-ligation, indicating the duct’s ability to proliferate and induce tissue repair (Walker and Gobe, 1987). Takahashi et al. indicated that precursor acinar cells, which are primarily produced from ductal cells, are the primary source of new acinar cells in atrophic glands (Takahashi, et al., 2004b). Subsequent expansion of acinar cells depends on the proliferation and maturation of the newly formed cell. Cotroneo et al. found that branch-like ductal structures emerged during gland regeneration, similar to the development of glands in the embryonic stages, and the localisation of SMG-B protein from ductal cells to acinar cells during regeneration further demonstrated that the newly developed acinar cells were derived from ductal cells after de-ligation (Cotroneo, et al., 2008).

Furthermore, myoepithelial cells play an important role in gland regeneration (Thiemann, et al., 2022). Myoepithelial cells are present on the basement membrane of all exocrine glands and have been shown to exhibit stem/progenitor cell-like functions in a number of organs, including the submucosal glands of lung and the mammary gland (Prater, et al., 2014; Tata, et al., 2018). Myoepithelial cells represent a self-sustained cell lineage during salivary gland development and adulthood which show strong cellular flexibility during salivary gland regeneration (May, et al., 2018; Song, et al., 2023). Takahashi et al. have reported that the presence of proliferating myoepithelial cells covering newly formed acini during regeneration of SMG after duct de-ligation (Takahashi, et al., 2004a). Ninche et al. hold the view that more than 80% of regenerated acini originate from differentiated cells, including ductal and myoepithelial cells, during the regeneration of salivary glands after duct ligation injury. They demonstrated that myoepithelial cells are involved in tissue regeneration by reverting to a bipotent progenitor cell state and subsequent redifferentiation into acinar cells after duct ligation (Ninche, et al., 2020).

Recently, salivary gland epithelial stem/progenitor cells have been recognised as the predominant cell types that promote gland regeneration (Lombaert, et al., 2011; Song, et al., 2024). Characteristics of adult stem cells are observable during rodent gland regeneration (Barrows, et al., 2024). Kwak and Ghazizadeh suggested that basal cells of the excretory and intercalated ducts have the potential for infinite proliferation and directional differentiation and can be considered as salivary gland stem cells (Kwak and Ghazizadeh, 2015). CK5 is a marker of stem/progenitor cells in several tissues and is a basal epithelial cell marker in adult salivary glands; acetylcholine signaling could increase the CK5^+^ salivary gland epithelial progenitor cell population to promote epithelial morphogenesis and epithelial organ regeneration (Knox, et al., 2010; Knox, et al., 2013). In homeostatic conditions, CK5^+^ ductal cells do not give rise to new acinar cells, whereas after the release of duct ligation, they can generate acinar cells to promote tissue regeneration (Weng, et al., 2018). CK14 has been proven to be a marker of ductal progenitor populations in murine salivary glands (Narendra, et al., 2023). Kwak et al. showed that CK14-expressing excretory and intercalated ductal cells in the adult mouse SMG contribute only to ducts but not to acinar cells under homeostatic conditions (Kwak, et al., 2016). May et al. showed that CK14^+^ progenitor cells are fast-cycling cells that multiply in response to radiation-induced damage by persistent proliferation, and repopulate the granulated ducts by asymmetric division (May, et al., 2018). Zhang et al. demonstrated that intra-flagellar transport (IFT^+^)/CK14^+^ stem/progenitor cells can promote gland regeneration by self-renewing and differentiating into granular ductal cells after duct de-ligation (Zhang, et al., 2022). Hisatomi et al. demonstrated that SMG duct ligation can induce the proliferation and expansion of Sca-1^+^/c-Kit^+^ stem cells to repair injury (Hisatomi, et al., 2004). Subsequently, Lombaert et al. successfully isolated and cultured Sca-1^+^/c-Kit^+^ stem cells from murine SMG and confirmed that stem cells were derived from ductal cells. *In vitro,* these cells differentiated into acinar cells, which produce mucin and amylase, as well as ductal cells. *In vivo,* the intra-glandular transplantation of a small number of c-Kit^+^ cells led to the sustained restoration of the structure and function of salivary glands (Lombaert, et al., 2008). Moreover, Ninche et al. revealed that c-Kit^+^ ductal cells can differentiate into acinar cells after mouse SMG duct ligation. Najafi et al. isolated and cultured mesenchymal stem cells (MSCs) from mouse SMG and demonstrated that local injection has the potential for reconstructing the full histological structure of necrotic tissue after duct ligation (Najafi, et al., 2020; Marinkovic, et al., 2023). Additionally, the transcription-related factor Ascl3 identifies an adult progenitor cell involved in the development and maintenance of salivary glands (Yoshida, et al., 2001; Bullard, et al., 2008). Ascl3-expressing cells located in ducts are active proliferating progenitors, which were also able to produce new acinar cells in a duct ligation/de-ligation model (Arany, et al., 2011).

The transcription factor Sex determining region Y-box 2 (Sox2) preserves the pluripotency of early embryonic cells and mediates the generation of various epithelia throughout foetal development. It has been demonstrated that Sox2 is a progenitor cell marker in the SMG and sublingual glands (SLG) of foetal mice, which primarily gives rise to acinar cells rather than ductal cells during salivary gland homeostasis (Arnold, et al., 2011). Emmerson et al. suggested that muscarinic signaling is essential for regulating Sox2-mediated replacement of acinar cells, targeting Sox2^+^ cells and preserving muscarinic signaling may help restore salivary function after radiation damage (Emmerson, et al., 2018). However, the role of Sox2 in the regeneration of salivary gland duct ligation/de-ligation has not been investigated. Mist1 is a specific marker for acinar cells, and its expression begins at the early stage of cell differentiation and continues into adulthood (Pin, et al., 2000). Mist1^+^ acinar cells could proliferate and extend to promote gland regeneration after duct ligation/de-ligation (Aure, et al., 2015b). Thus, the damaged-salivary glands rely on the remaining surviving cells and recruit differentiated cell populations, or stem/progenitor cells to guarantee secretory cell renewal and regeneration. Further studies should aim to provide greater clarity regarding the potential cell populations responsible for gland regeneration using this model.

### 3.3 Signaling pathways of salivary gland duct de-ligation

**FIGURE 4:**
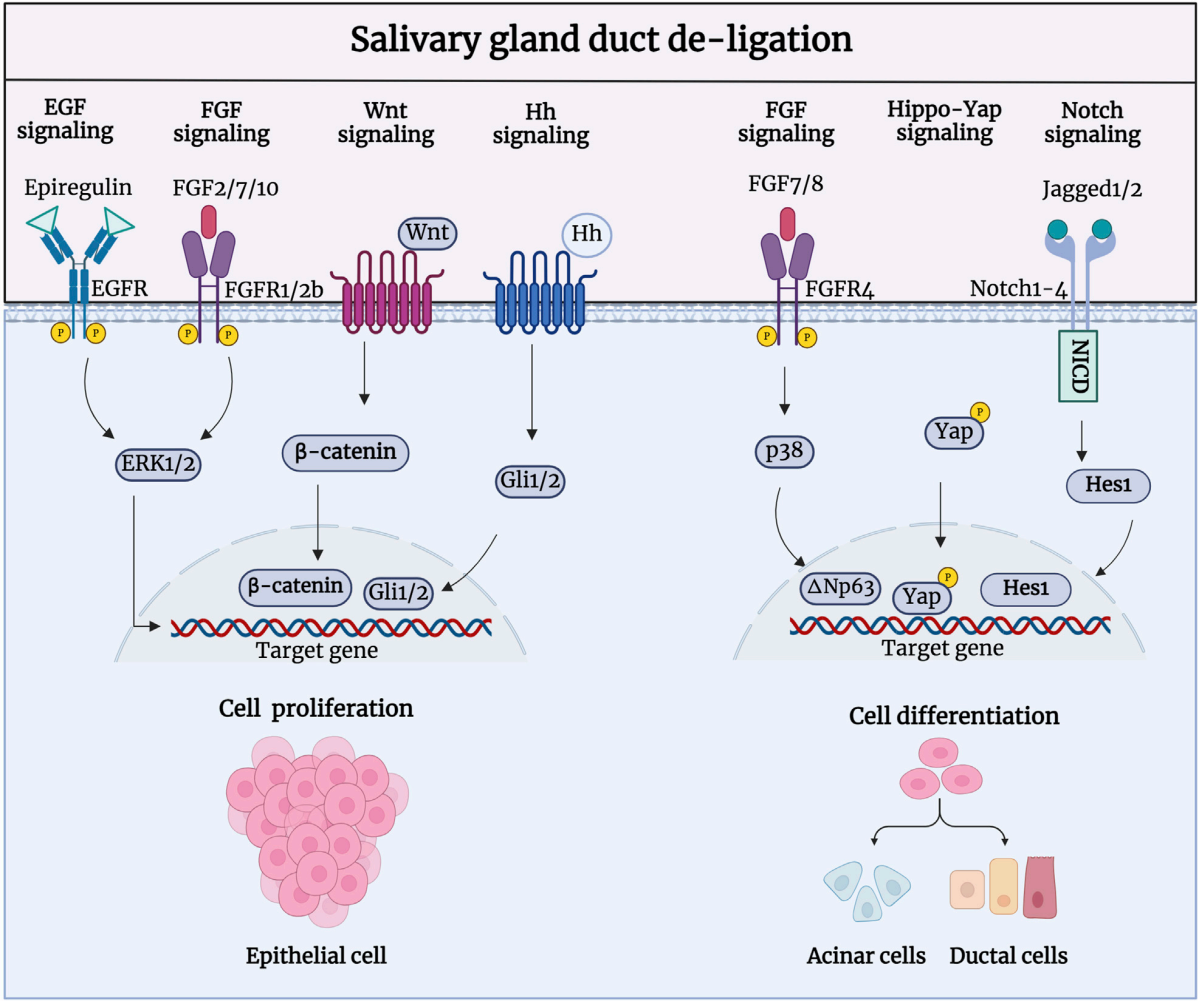
Signaling pathways involved in the regeneration process after salivary gland duct de-ligation. During salivary gland regeneration, multiple signaling pathways, including FGF, EGF, Notch, Hedgehog, and Wnt/β-catenin, are activated to regulate salivary gland regeneration. FGF2/7/10-/FGFR1/2b, Epiregulin/EGFR, Wnt/β-catenin, and Hh/Gli are responsible for the acinar and ductal cell proliferation to promote regeneration. FGF7/8-FGFR4, Jagged1/2-Notch1-4, and Hippo-Yap are responsible for cell differentiation to generate acinar and ductal cells during the regeneration process. Figure was created with BioRender.com.

During salivary gland regeneration, multiple signaling pathways, including fibroblast growth factor (FGF), epidermal growth factor (EGF), Notch, Hedgehog, and Wnt/β-catenin are activated to regulate cell proliferation (Figure 4). Shimizu et al. showed that FGF signaling is highly active during regeneration. After duct ligation in their study, there was a significant increase in the expression of FGF-2, FGF-7, FGF-8, and FGF-10 in duct-like epithelial cells and newly formed acinar cells, as well as a significant increase in FGFR1 and FGFR4 in intercalated ducts, were significantly increased (Shimizu, et al., 2015). Therefore, FGFs may facilitate gland regeneration by promoting the formation of new acinar cells through intercalated ducts. Further research has shown that FGF7-FGFR2 signaling drives the activity of ductal progenitor differentiation and maturation of acinar cell structure, via MAPK signaling in the SMG and SLG (Steinberg, et al., 2005; Aure, et al., 2023). Zaia et al. demonstrated that retrograde transduction of bFGF post-duct de-ligation stimulates acinar and ductal cell proliferation, promoting regeneration in rat SMG (Okazaki, et al., 2000). bFGF is a highly bioactive peptide growth factor that promotes cell proliferation in the G0 and G1 phases (Liu, et al., 2021; Huang, et al., 2023). Furthermore, the EGF signaling pathway has been shown to be activated during gland regeneration; the SMG is considered the most important organ for circulating EGF production (Tsutsumi, et al., 1986; Cho, et al., 2021). Nagai et al. reported a significant increase in the expression of epithelial regulatory proteins (epiregulin), heparin-conjugated epidermal EGF (HB-EGF), and epidermal growth factor receptors (EGFR). Epiregulin, a proliferative factor in ductal cells, enhances binding to EGFR in ductal epithelial cells and branch-like acinar structures to induce autocrine or paracrine secretion of the EGF family feedback loop, stimulating tissue repair (Nagai, et al., 2014). *In vitro* experiments have shown that the isolation and culture of salivary gland epithelial cells from ligated glandular tissue produced a large number of EGF ligands, and their proliferative ability was significantly enhanced after stimulation with epiregulin.

Activation of extracellular signal-regulated kinase (ERK) drives cell proliferation, which could be achieved by FGF and EGF binding to receptors such as FGFRs and EGFRs (Wang, et al., 2021a). Dang, et al., 2008 was the first to discover that MAPK signaling was activated to regulate cell proliferation during the regeneration of PG duct ligation. A recent study showed comparable expression patterns of ERK1/2 and p38, which were significantly upregulated 7 days after rat PG duct de-ligation. Phosphorylated ERK1/2 is predominantly expressed in the intercalated and striated ducts, and phosphorylated p38 is mainly present in the intercalated ducts striated ducts, and stroma cells. ERKs have traditionally been linked to cell proliferation, while p38 is constantly involved in the regenerative differentiation of epithelial cells (Zhuang, et al., 2005). The activation of ERKs upon duct obstruction is consistent with increased signaling via upregulated EGFR, which may also indicate that the activation of ERKs may be caused by an increase in EGFR. In addition, the activation of FGF10-induced ERK1/2 phosphorylation could promote proliferation in human PG cells (HSY) and regulate branching morphogenesis in the salivary epithelium (Yamada, et al., 2016).

Hai, et al., 2010 showed that both Wnt/β-catenin and Hh signaling were activated during gland development and regeneration after duct de-ligation. During embryonic SMG development, Wnt and Hh signaling promote cellular polarisation and acinar cavity formation in developing glandular epithelial cells (Patel, et al., 2011; Guan, et al., 2019). Wnt/β-catenin activity is decreased in the mature salivary gland but is significantly enhanced in the proliferation of intercalated ducts during glandular regeneration after duct de-ligation (Xu, et al., 2022). Activated Wnt/β-catenin signaling can trigger the transmission of Hh signaling and subsequently promote the proliferation of epithelial stem/progenitor cells to regulate gland regeneration. Wnt and Hh signaling perform biological functions in a synergistic or interdependent manner. Wnt signaling can directly activate the expression of Gli2 to promote cell proliferation (Borycki, et al., 2000; Pantazi, et al., 2017). The transcriptional regulator Yes-associated protein (YAP), a downstream effector of Hippo signaling pathway, is a vital facilitator of tissue development and regeneration in many different organs (Yui, et al., 2018). Nuclear-localised YAP, that is restricted to the acinar and intercalated duct compartments, is known to be involved in gland preservation, which is present in low quantities during salivary gland homeostasis (Kim, et al., 2008; Chibly, et al., 2014; Szymaniak, et al., 2017). After duct de-ligation, there is a noticeable and region-specific increase in YAP-expressing cells which switch to a stem-like cell state and drive the salivary gland regeneration program, confirming a role for Hippo signaling pathway inhibition in the response to injury (Rocchi, et al., 2021).

As a member of the p53 family, transcription factor p63 (ΔNp63) plays an essential role in the development, homeostasis, and regeneration of epithelial tissue (Weiner, et al., 2022; Song, et al., 2023). Ikai et al. have shown that the expression of ΔNp63, which is mainly located in α-SMA^+^ myoepithelial cells and CK5^+^ cells, significantly increased after duct de-ligation, indicating that the proliferation ability of these cells is enhanced during PG regeneration. FGF7 can also regulate the phosphorylation of p38 and further upregulate the expression of ΔNp63 to promote the cell differentiation and gland generation (Ikai, et al., 2020). ΔNp63 regulates cell proliferation and differentiation by activating Jagged1 through Notch signaling, suggesting that the Notch signaling pathway may be implicated in the salivary gland regeneration (Ma, et al., 2010). Dang et al. showed that the expression levels of Notch 1–4, Notch ligands Jagged 1 and 2, and Hes-1 were elevated in the atrophic gland after rat PG duct ligation and progressively recovered to baseline levels following duct de-ligation. The nuclear labelling of Notch ligand and receptor in the regenerated glands revealed their involvement in acinar cell differentiation (Dang, et al., 2009; Wang, et al., 2021c). Additionally, Ahn et al. showed that the activity and mRNA expression levels of the purine receptor P2Y2 were significantly altered at different times after duct de-ligation (Ahn, et al., 2000; Jasmer, et al., 2021). In conclusion, many signaling pathways are activated after duct de-ligation and may be interconnected to regulate epithelial cell proliferation, cell differentiation, and tissue regeneration. The signaling pathways involved in duct de-ligation are intimately connected; nonetheless, we examined each pathway independently to determine which targets are involved in the process of injury and regeneration to provide more precise recommendations for the treatment of salivary gland dysfunction.

## 4 Fibrosis in salivary gland duct ligation/de-ligation

**FIGURE 5:**
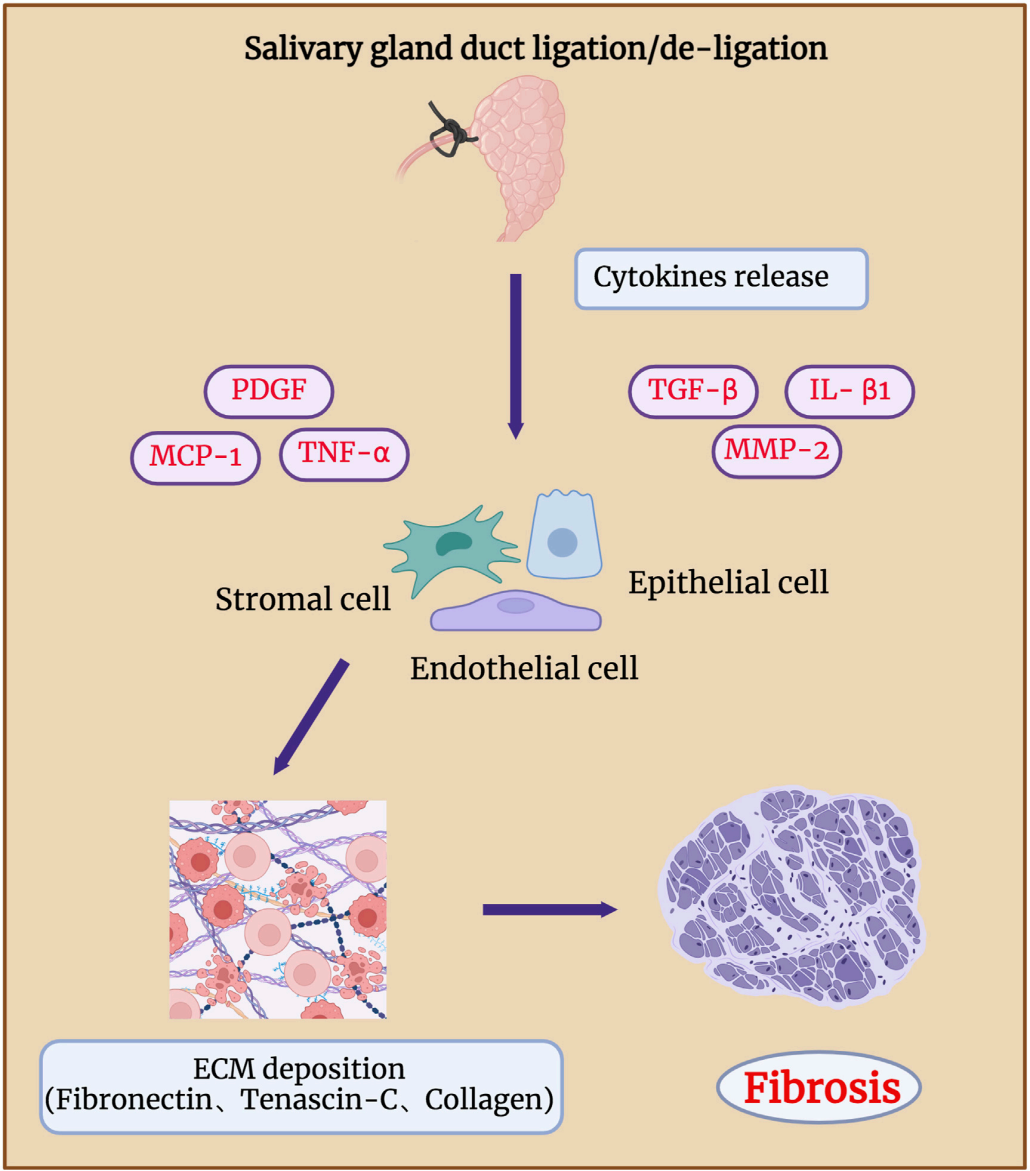
Fibrosis in salivary gland after duct ligation/de-ligation. Chronic salivary gland injury can increase collagen deposition in the intralobular regions, eventually leading to glandular atrophy and fibrosis. Duct ligation leads to an increase in the expression of genes related to fibrosis, such as TNF-α, IL-β1, MMP-2, PDGF, TGF-β, and MCP-1. These genes drive the production of ECM by epithelial, endothelial, and stromal cells, which in turn leads to glandular fibrosis. Figure was created with BioRender.com.

Long-term duct ligation can increase collagen deposition in the septum, intralobular regions, and elastic fibres around the ducts, eventually leading to glandular atrophy and fibrosis (Zaia, et al., 1996; Zhang, et al., 2020) (Figure 5). Hishida et al. showed that damage to the nerve fibres around the duct may lead to ligation-associated gland atrophy. The binding of substance P-positive nerve fibres (SPFs) distributed around the ducts and acinar substance P receptors (SPRs) localised in the cytoplasm of ductal cells can regulate the repair of glands after duct de-ligation, whereas inhibition of SPRs by injection of antagonists cause obvious acinar atrophy. Aberrant TGF-β signaling is reportedly involved in salivary gland fibrosis (Sisto, et al., 2021; Li, et al., 2023). TGF-β is expressed in clinical samples of people with chronic obstructive sialadenitis (Kizu, et al., 1996; Teymoortash, et al., 2003). Yasumitsu, et al., 2018 demonstrated that TGF-β signaling was significantly activated after SMG duct de-ligation. Woods et al. showed that upregulated TGF-β signaling is associated with the occurrence and regression of glandular fibrosis during duct ligation/de-ligation, and the expression of TGF-β1, -β3, and βR1, but not -β2 increases robust after mice salivary gland duct ligation (Woods, et al., 2015). TGF-β/TGF-βR activated SMAD and E-cadherin to regulate collagen deposition and glandular fibrosis (Hall, et al., 2010). The use of an exogenous TGFβR1 inhibitor decreased the levels of fibrotic markers in the mouse model of ligation-induced salivary gland injury (Wang, et al., 2023b). These findings align with TGF-β signaling in liver fibrosis models generated by bile duct obstruction (Sun, et al., 2022).

According to Ueda, et al., 2009, extracellular matrix components, such as fibronectin and tenascin-C, mainly affect the formation of duct-like acinar structures during glandular regeneration after duct de-ligation, whereas collagen types III and IV are mainly involved in the acinar cell regeneration. Activation of receptor tyrosine kinase-like orphan receptor (Ror)-mediated signaling after duct ligation may also be associated with glandular fibrosis. The expressions of Ror2, Ror1, Wnt5a, and fibrosis-related genes such as TNF-α, IL-β1, and MMP-2 were significantly increased 7 days after mice SMG duct ligation (Takahashi, et al., 2017). Mao et al. revealed that the function of the endothelial barrier is compromised during the process of mouse SMG duct ligation-induced fibrosis. The deterioration of the epithelial barrier function in fibrotic glands is caused by the loss of claudin-5 at the endothelial tight junction areas, which is most likely brought on by monocyte chemoattractant protein-1 (MCP-1) (Cong, et al., 2018; Mao, et al., 2023). Amber et al. found that collagen actively remodelling and extracellular matrix accumulation both markedly increased 14 days after mouse SMG duct ligation (Altrieth, et al., 2023b). They analysed the population of all cell types after duct ligation and showed that Gli1^+^, Pdgfra^+^, and Pdgfrb^+^ stromal cells are involved in extracellular matrix deposition, which indicates that Pdgfr^+^ and Gli1^+^ stromal fibroblasts are potentially in charge of producing extracellular matrix (ECM) and glandular fibrosis (Altrieth, et al., 2023a). Therefore, to reveal potential therapeutic targets, future studies should focus on identifying the signaling pathways that cause fibrotic responses in epithelial, endothelial, and stromal cell subtypes.

## 5 Parasympathetic innervation in the salivary gland duct ligation/de-ligation

**FIGURE 6:**
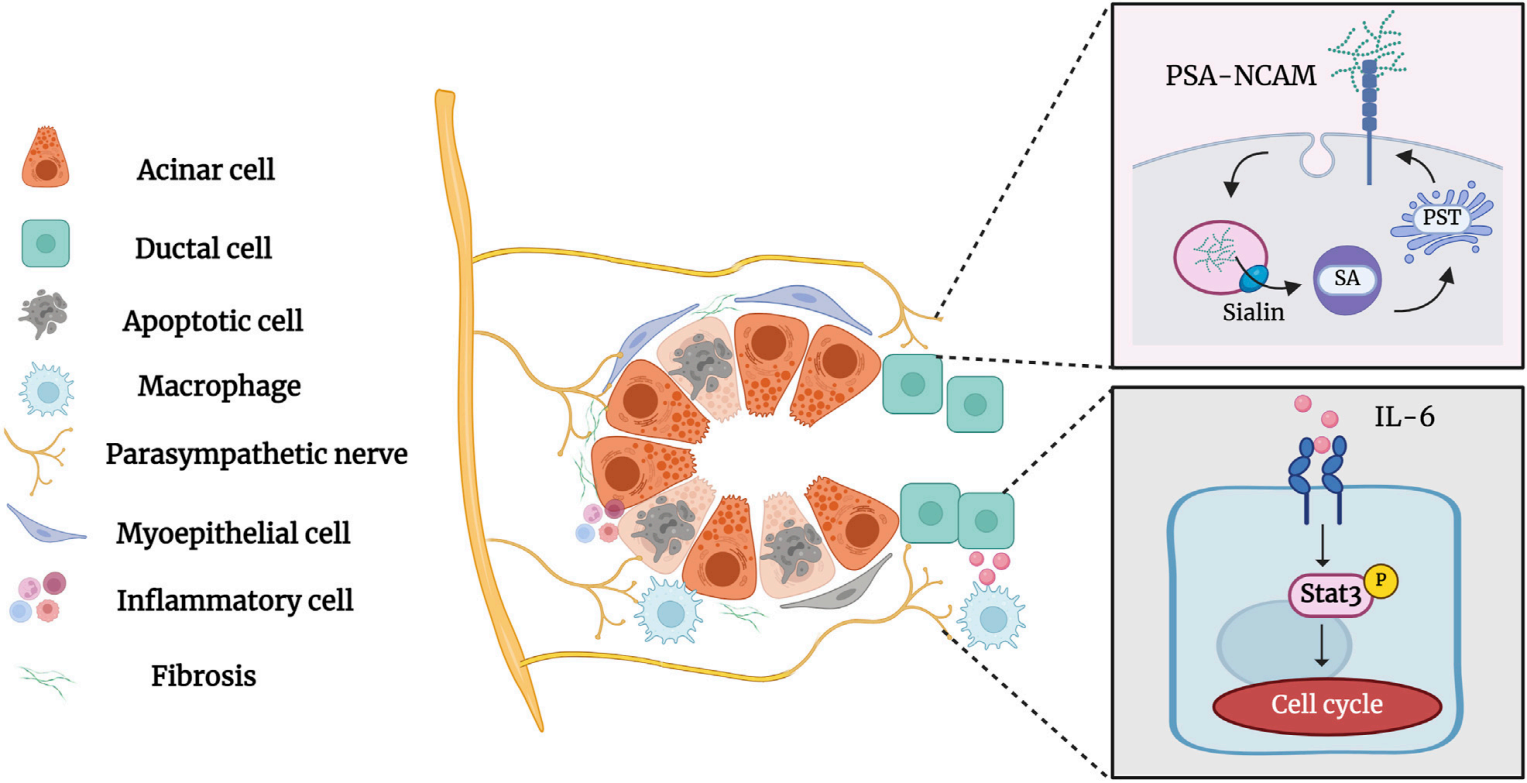
Parasympathetic innervation in the salivary gland after duct ligation/de-ligation. Parasympathetic innervation influences glandular regeneration after duct ligation/de-ligation. The intact parasympathetic nerve promotes SMG regeneration through ductal cell proliferation, which is mainly associated with increased expression of PST and NCAM. In addition, the parasympathetic-macrophage-ductal epithelial cell axis promotes SMG regeneration after duct ligation/de-ligation by activating IL-6/STAT3 signaling pathways. Figure was created with BioRender.com.

Parasympathetic innervation occurs concurrently with salivary gland development and is essential for the activation of epithelial progenitor cells during organogenesis (Nedvetsky, et al., 2014; Shindo, et al., 2022). Parasympathetic innervation reportedly correlates with gland acinar area and secretory function (Proctor and Carpenter, 2014). Zhang, et al., 2019 demonstrated that parasympathectomy results in weight loss, glandular atrophy, and fibrosis. Osailan, et al., 2006 showed that parasympathectomy causes severe inflammatory cell infiltration and atrophy following duct ligation. The interaction between the parasympathetic nerve and the salivary glands is essential for maintaining neuron-epithelial communication. Tissue repair and cell regeneration are impossible in the absence of elements that preserve neuronal-epithelial connection and functional innervation (Knox, et al., 2013). Wang et al. showed that complete parasympathetic innervation promotes SMG regeneration after rat duct ligation/de-ligation, whereas parasympathectomy considerably prevents the restoration of gland structure and function. Maintaining parasympathetic innervation in SMG is associated with the increased duct proliferation and elevated expression of polysialyltransferase IV (PST) and neural cell adhesion molecules (NCAM) (Carpenter, et al., 2009; Wang, et al., 2021b). PST is a glycosyltransferase that synthesises sialic acid polymers, which play an important role in salivary gland regeneration (Colley, et al., 2014). After duct ligation, the expression of the sialic acid transporters sialin, polysialic acid (PSA), and PST increases significantly, indicating that sialin may regulate the regeneration of the SMG by affecting PSA synthesis (Wang, et al., 2022). Subsequent studies by Wang et al. showed that the parasympathetic-macrophage-ductal epithelial cell axis promotes SMG regeneration in female rats after excretory duct ligation/de-ligation. Parasympathetic innervation triggers IL-6 release by regulating macrophage paracrine activity via muscarinic signaling, subsequently activating STAT3 to promote epithelial proliferation and SMG regeneration (Wang, et al., 2023a). The role of parasympathetic innervation in the development and regeneration of the salivary glands cannot be ignored (Figure 6). However, the specific mechanism of parasympathetic innervation in regeneration after salivary gland duct ligation/de-ligation remains unclear, and future studies are needed to explore additional helpful information involved in the process.

## 6 Conclusion

Salivary gland duct ligation/de-ligation models are an excellent candidate model for studying the glandular atrophy and regeneration. Duct ligation/de-ligation involve several processes, including apoptosis and proliferation of salivary gland epithelial cells and myoepithelial cells, infiltration of inflammatory cells such as neutrophils and macrophages, and fibrosis due to the deposition of collagen fibres. The activation of signaling involved in the processes of injury and regeneration is complex. Furthermore, parasympathetic innervation plays a role in regulating the injury and regeneration of salivary glands. Given that the salivary glands of rodents employed in animal studies displayed anatomical physiologies that are comparable but distinct from those of humans, there is still uncertainty about whether findings in animal models could have an equivalent impact on humans. Therefore, it is essential to create and use animal models that more closely match and simulate the pathologic circumstances of humans. More appropriate animal model systems would be a great opportunity to advance our knowledge of mechanisms that are both reparative and destructive in both normal and pathological conditions. Additional animal and clinical studies in the future should be conducted to clarify the molecular mechanisms of salivary gland injury and regeneration, thereby discovering more effective treatment approaches.

Currently, oral lubricants, saliva substitutes, and muscarinic agonists continue to be the main palliative therapies for patients with salivary gland hypofunction (Chibly, et al., 2014). However, the benefits are short-lived, and their capacity to alleviate severe hypofunction is not always consistent. Long-term use of pilocarpine, a muscarinic agonist, can result in major adverse effects like dizziness, nausea, stomach pain, and blurred vision (Barbe, 2018). Therefore, new therapeutic strategies need to be explored. Based on these current findings presented in this review, some strategies have been used to rescue salivary gland dysfunction in animal models and humans. There is rising interest in employing small-molecule inhibitors that target receptor kinases or inflammatory pathways such as NF-κB signaling. Specific monoclonal antibody anti-CD20 (rituximab), which inhibits NF-κB signaling, may improve the effect of treatment (De Vita S, et al., 2014). Furthermore, preclinical models have demonstrated that blocking the ATP-gated P2X7 receptor enhances saliva production and decreases lymphocytic infiltration in a mouse model of SS. A variety of anti-fibrotic therapeutic strategies have been developed, such as targeting TGF signaling. The TGF-β pathway has several possible targets, including anti-fibrotic ligand/receptor activation, pro-fibrotic ligand/receptor activity inhibition, and SMAD signaling inhibition (Zhang, et al., 2020). Wang et al. indicated that metformin exerts anti-fibrotic effects by inhibiting the phosphorylation of SMAD2/3 in a rat duct ligation model and human chronic salivary gland inflammation (Wang, et al., 2023b). In addition, human keratinocyte growth factor-1 (FGF7), IGF, and neurturin gene therapy via retroductal injection of the murine salivary gland could prevent radiation-induced salivary hypofunction. Lombaert et al. demonstrated that the morphology and function of the gland were recovered after radiation by intraglandular transplantation of 300 c-Kit^+^ cells isolated from salispheres into the recipient mouse SMG (Lombaert, et al., 2008). Similarly, salisphere-derived cells that express c-Kit with CD24 and CD49f increase the population of ductal and stem cells and reduce fibrosis (Nanduri, et al., 2011; Nanduri, et al., 2013). In summary, the therapeutic strategies have not been fully studied, and further research is needed to identify other potential targets to prevent injury, overcome fibrosis, and regenerate functional tissue. In the future, accumulated findings will facilitate the ability of clinicians to improve palliative treatments and provide permanent treatments.
